# Clinical Tests Predicting On-Road Performance in Older Drivers with Cognitive Impairment

**DOI:** 10.1177/00084174221117708

**Published:** 2022-08-10

**Authors:** Sarah Krasniuk, Alexander M. Crizzle, Ryan Toxopeus, Diane Mychael, Natasha Prince

**Keywords:** Automobile driving, Cognition disorders, Comprehensive driving evaluation, Trails B, Useful Field of View®, Conduite automobile, évaluation complète de la capacité de conduire, Trails B, troubles cognitifs, Useful Field of View^MD^

## Abstract

**Background.** The Trail Making Test Part B (Trails B) and Useful Field of View® (UFOV) can predict on-road outcomes in drivers with cognitive impairment (CI); however, studies have not included drivers referred for comprehensive driving evaluations (CDEs), who typically have more severe CI. **Purpose.** We determined the predictive ability of Trails B and UFOV on pass/fail on-road outcomes in drivers with CI (Montreal Cognitive Assessment <26) referred for CDEs. **Method.** Retrospective data collection from two driving assessments centers (*N*  =  100, mean age  =  76.2  ±  8.8 years). **Findings.** The Trails B (area under the curve [AUC]  =  .70) and UFOV subtests 2 (AUC  =  .73) and 3 (AUC  =  .76) predicted pass/fail outcomes. A cut-point ≥467 ms on UFOV subtest 3 better-predicted pass/fail outcomes with 78.9% sensitivity and 73.5% specificity. In comparison, a cut-point ≥3.58 min on Trails B had lower sensitivity (73.7%) and specificity (61.8%). **Implications.** The UFOV subtest 3 may be more useful than the Trails B for predicting pass/fail outcomes in drivers with more severe CI referred for CDEs.

## Résumé

**Description.** Les évaluations Trails B et Useful Field of View^MD^ (UFOV) peuvent prédire les résultats sur route des conducteurs ayant un déficit cognitif; cependant, les études réalisées n’incluaient pas les conducteurs soumis à une évaluation complète de leur capacité de conduire, qui présentent typiquement un déficit cognitif plus aigu. **But.** Nous avons déterminé l’aptitude de Trails B et d’UFOV à prédire la réussite ou l’échec des conducteurs présentant un déficit cognitif (MoCA <26) et soumis à une évaluation complète de leur capacité de conduire. **Méthodologie.** Collecte de données rétrospective auprès de deux centres d’évaluation de la conduite automobile (*N*  =  100, âge moyen  =  76,2  ±  8,8 ans). **Résultats.** Le test Trails B (SSC  =  0,70) et les sous-tests UFOV 2 (SSC  =  0,73) et 3 (SSC  =  0,76) ont permis de prédire la réussite ou l’échec. Un point de découpage ≥467 millisecondes pour le sous-test 3 de l’UFOV prédisait mieux le résultat, avec une sensibilité de 78,9% et une spécificité de 73,5%. En comparaison, un point de découpage ≥3.58 min pour le test Trails B présentait une sensibilité (73,7%) et une spécificité (61,8%) plus faibles. **Conséquences.** Le sous-test 3 de l’UFOV pourrait être plus utile que le test Trails B pour prédire la réussite ou l’échec à une évaluation complète de la capacité de conduire chez les conducteurs présentant un déficit cognitif plus aigu.

## Introduction

Cognitive impairment (CI) can arise due to different medical conditions, such as dementia, for which the prevalence often increases with age (Fiest et al., 2016; Public Health Agency of Canada, 2020). However, the manifestation of CI in terms of driving behavior can depend on the stage of illness among other factors. For example, those with early dementia, or mild CI (MCI), may experience problems with attention, executive function, information processing, memory, and/or visuospatial ability, which can compromise their ability to drive safely (Rashid et al., 2020). Hence, it is critical that clinicians use assessments that have the sensitivity and specificity to pick up on subtle yet important differences in CI that can affect behind-the-wheel behavior.

Research studies show that up to 70% of older adults with CI fail the on-road assessment (Carr et al., 2011; Chee et al., 2017; Eramudugolla et al., 2021; Fuermaier et al., 2019; Hird et al., 2016; Jacobs et al., 2017; Piersma et al., 2018; Toepper & Falkenstein, 2019). Older adults with CI have poorer speed and lane control and route-following behaviors than healthy controls, and perform worse on the on-road assessment, particularly when changing lanes, merging, navigating, and identifying landmarks or signs (Barco et al., 2015; Chee et al., 2017; Eramudugolla et al., 2021; Fuermaier et al., 2019; Griffith et al., 2013; Hird et al., 2016; Jacobs et al., 2017; Toepper & Falkenstein, 2019; Wadley et al., 2009).

Clinicians use a variety of assessments to identify whether drivers are medically fit or unfit to continue driving (Gibbons et al., 2017), especially in older drivers with CI (Stern et al., 2016). Two prominent assessments are the Trail Making Test Part B (Trails B), a measure of divided attention, and the Useful Field of View® (UFOV), a measure of visual processing speed (i.e., subtest 1), divided attention (i.e., subtest 2), and selective attention (i.e., subtest 3) (Gentzler & Smither, 2012). Numerous systematic reviews show that the Trails B and UFOV are associated with on-road driving performance in older drivers (Dickerson et al., 2014; Duncanson et al., 2018; Mathias & Lucas, 2009; Seong-Youl et al., 2014; Vrkljan et al., 2011), as well as drivers with Parkinson's disease (Classen et al., 2015; Crizzle et al., 2012a), multiple sclerosis (Fragoso et al., 2016; Krasniuk et al., 2019), traumatic brain injury (Egeto et al., 2019), stroke (Babulal et al., 2020; Devos et al., 2011), MCI (Hird et al., 2016; Withaar et al., 2000), and dementia (Bennett et al., 2016; Hird et al., 2016; Molnar et al., 2006; Rashid et al., 2020; Withaar et al., 2000). However, only a few studies have examined the predictive ability with associated cut-points of the Trails B and UFOV in predicting pass/fail on-road outcomes in older adults with CI (Bowers et al., 2013; Dobbs & Shergill, 2013; Duncanson et al., 2018; Papandonatos et al., 2015; Roy & Molnar, 2013; Stern et al., 2016).

A prior systematic review shows support for using the cut-point of ≥3 min (Betz & Fisher, 2009; Classen et al., 2008; Staplin et al., 2003; Roy & Molnar, 2013) or ≥3 errors (Mazer, Korner-Bitensky, & Sofer, 1998) on the Trails B although other cut-points were identified including ≥90 s (Hargrave et al., 2012), ≥133 s (Marottoli et al., 1998), and ≥147 s (Ball et al., 2006); however, these studies were not specific to older adults with CI or included on-road testing (Roy & Molnar, 2013). In a sample of older drivers (*n*  =  134; 35.1% with CI), Dobbs and Shergill (2013) found that using the 3 min cut-point on the Trails B had good specificity (88%) but poor sensitivity (50%) and suggested a cut-point of ≥140.5 s on the Trails B, which suggested more balance between specificity (77%) and sensitivity (77%). Papandonatos et al. (2015) compared the ability of the Trails B to predict on-road outcomes in two samples of older drivers with CI and found that more than 25% of participants were unable to complete the Trails B within 5 min. A cut-point of ≥108 s was suggested to distinguish between unsafe versus safe/marginal drivers. In both samples, the sensitivity was 88%; however, the specificity was 21% and 40%, respectively (Papandonatos et al., 2015). In another study, Duncanson et al. (2018) found that the Trails B cut-point of ≥138 s was not sensitive (i.e., 60% sensitivity, area under the curve [AUC]  =  .63) in predicting failing outcomes in drivers aged 65 and older with CI.

Prior studies show there is an association between UFOV scores (subtests and Risk Index) and on-road driving performance in those with CI (Bowers et al., 2013; Stern et al., 2016). For example, Bowers et al. (2013) found that in a clinical battery of visual and cognitive tests, the UFOV subtest 2 was the best single predictor of identifying “at-risk” (8 of 18 drivers had MCI) versus “safe” older drivers (3 of 29 drivers had MCI) with a cut-point of 191.50 ms and a 72% specificity and 93% sensitivity (AUC  =  .84, 95% CI  =  [.72, .97]). Stern et al. (2016) found that the UFOV subtest 2 score discriminated between older “at-risk” drivers and “not at-risk” drivers (*M*  =  360 ms, *SD*  =  139 vs *M*  =  154 ms, *SD*  =  146, *p* < .001) in a mixed sample of 44 healthy drivers, 20 with MCI, and 20 with dementia. However, these prior studies had sample sizes that were both small and of mixed composition (Bowers et al., 2013; Stern et al., 2016), precluding the ability to determine whether the UFOV is predictive of driving impairment in older drivers with CI.

Prior driving studies often rely on volunteers; however, volunteers tend to be healthier, more educated, and confident in their behind-the-wheel abilities (Crizzle et al., 2012b). Additionally, prior studies have included drivers with CI that vary in their medical condition (e.g., MCI, Alzheimer's disease, and dementia), level of impairment, and symptomology, which is expected among volunteer participants (Fuermaier et al., 2019; Hird et al., 2016; Piersma et al., 2018). Drivers with more significant impairments are reluctant to participate in research studies due to fear of losing their driver's license (Crizzle et al., 2012b). By failing to include drivers with CI with significant driving difficulties identified by physicians and referred for a comprehensive driving evaluation (CDE), it is difficult to determine the true discriminability of clinical tests to screen for unsafe driving. The purpose of this study is to determine the predictive ability of the Trails B and UFOV in drivers with CI referred by physicians to undergo a CDE. The objectives are: (1) to compare the Trails B and UFOV in predicting pass/fail on-road outcomes in a sample of drivers with CI referred for a CDE and (2) to determine optimal cut-points along with their sensitivity, specificity, positive and negative predictive values, misclassifications, and error rates.

## Methods

### Study Design

This study received ethics approval from two Canadian Universities. Data from CDEs, administered by an occupational therapist, were collected retrospectively from one driving assessment center in Southwestern Ontario and from one driving assessment center in Saskatchewan. The occupational therapists were trained and experienced in assessing fitness to drive via performing CDEs; one was a certified driver rehabilitation specialist and the other received advanced training through a graduate certificate in Assessing Driving Capabilities. The CDEs consisted of information about the driver's medical status (e.g., diagnoses and comorbid medical conditions), driving history (e.g., license status and number of years driving), and scores on a clinical battery of tests (e.g., vision, motor control, and cognition) followed by an on-road assessment. The occupational therapists in Ontario and Saskatchewan administered both the clinical and on-road assessments.

### Participants

In total, 201 drivers from Southwestern Ontario and 183 drivers from Saskatchewan were referred for a CDE due to medical concerns or conditions (*n*  =  314; e.g., age-related declines, alcoholism, amputation, aphasia, brain injury, cognitive declines, CI, complex regional pain syndrome, concussion, diabetes, bipolar disorder, language deficits, posttraumatic stress disorder, motor vehicle crash involvement, multiple sclerosis, numbness and paresthesia, Parkinson's disease, pulmonary atresia, sleep apnea, spinal injury, stroke, and vision deficits), to renew an expired driver's license (*n*  =  1); to undergo a reassessment (*n*  =  38); to test driver controls or adaptive equipment (*n*  =  10); to test night driving (*n*  =  1); or the reasons were not stated (*n*  =  20). The inclusion criteria for this study required that drivers were referred and underwent the CDE; drivers had a diagnosis of CI; and drivers had a score of below 26 on the Montreal Cognitive Assessment (MoCA) (Canadian Task Force on Preventive Health Care, 2016; Nasreddine et al., 2005). There were 112 drivers with CI who underwent the CDE: 100 with scores below 26 on the MoCA. Accordingly, the study's final sample included 100 drivers (79 from Ontario and 21 from Saskatchewan).

### Data Collection and Procedures

**Clinical measures.** Data collected included demographic information (e.g., age, gender, education, location, and driving and health history) and scores on the MoCA, Trails B, and UFOV. The MoCA is a cognitive screening tool with scores ranging from 0 to 30; lower scores indicate worse cognitive functioning (Nasreddine et al., 2005). A cut-point of <26 on the MoCA has a sensitivity to detect up to 90% of patients with MCI and 100% of patients with mild Alzheimer's disease (Nasreddine et al., 2005).

The Trails B is a standardized paper and pencil test that measures a person's ability to divide attention between two competing tasks through connecting numbers and letters in ascending sequential order (Lezak, 1995). The test is timed in seconds and the number of errors is recorded. However, given the number of participants with missing error information, only the time to complete the Trails B was examined.

The UFOV is a computer-based test (PC model with touch screen) that includes three subtests that measure visual processing speed, divided attention, and selective attention, respectively (Visual Awareness Research Group, 2009). Subtest 1 requires a participant to identify a car or a truck that rapidly appears in the middle of a computer screen (visual processing speed). Subtest 2 also requires the participant to identify the car or truck, in addition to locating a symbol that appears in the periphery (divided attention). Subtest 3 requires the same tasks as in subtest 2, but while masking distracting objects displayed on the computer screen (selective attention) (Ball & Owsley, 1993). The score for each subtest indicates the participants’ response accuracy for 70% of stimuli presented on the screen, ranging from 0 to 500 ms. Using the combined performance on the three subtests, a Risk Index is calculated producing a score from 1 to 5, where 1 indicates a “very low risk” and 5 indicates a “high to very high risk” for driving performance impairment.

**On-road driving assessment.** The on-road driving assessment required participants to drive for 45 to 60 min, depending on traffic patterns, in a dual-pedal-equipped vehicle with automatic transmission. The on-road assessment was conducted in the daytime, in non-inclement weather conditions on residential, suburban, urban, and expressway sections. The occupational therapist sat in the passenger seat and assessed the participants’ ability to maneuver through the road course (95 total maneuvers). Pass/fail on-road outcomes were based on a scoring system where those who passed performed 47.5 or more total maneuvers correctly (or 50% or more maneuvers), and those who failed performed <47.5 total maneuvers correctly (or <50% of maneuvers) or they made a hazardous error such as running through a stop sign. Pass/fail outcomes were derived based on the occupational therapist's scoring system on the road assessment.

### Data Analysis

All data collected were entered into an SPSS database (IBM version 28.0) by graduate research assistants. Data entry was monitored by the research team to ensure data completion and accuracy. Data analyses were performed with SPSS (IBM version 28.0) using two-sided tests with a significance level of *p*≤.05. Summary statistics (i.e., continuous data: mean, standard deviation, and range; categorical data: frequencies and percentages) described participants’ demographic information (e.g., age, gender, education, location, and driving and health history), clinical test scores (i.e., MoCA, Trails B, and UFOV), and pass/fail outcomes on the on-road assessment. Pearson's *r* or rank biserial (*r*_rb_) correlations examined the bivariate correlations between age and clinical test scores. Independent sample *t*-tests or chi-square tests (χ^2^) examined differences in demographic or clinical test scores between those who obtained a pass/fail outcome on the on-road assessment.

A contrast receiver operating characteristic (ROC) curve was performed to discern the difference in the AUC between the Trails B (i.e., time for completion) and UFOV (i.e., subtests 1–3 and Risk Index) in predicting pass/fail outcomes. The AUC, an index of the overall predictive utility of a screening test, can range from 0.0 to 1.0 (perfect prediction) where .50 represents chance discrimination and .70 to .90 (*p* =.05) is considered an acceptable magnitude (Streiner & Cairney, 2007). For ROC curves plotted, we computed AUC estimates, 95% confidence intervals (95% CI), and *p*-values, and determined the sensitivity, specificity, positive and negative predictive values, misclassification, and error rate (1− sensitivity  +  1− specificity; false negatives  +  false positives) of selected cut-points. Youden's index (*J*), which indicates the highest discriminability between pass/fail outcomes when sensitivity and specificity have equal weight (*J* = sensitivity + specificity − 1), was calculated to determine optimal cut-points with the lowest error rates (Portney, 2020, pp. 521–523; Youden, 1950).

## Findings

### Participant Characteristics

The sample had a mean age of 76.2 years (*SD* = 8.8, range 45–94); 79% were men and 53.1% had more than a grade 12 education. MoCA scores ranged from 6 to 25 (*M* = 18.9, *SD* = 4.4). Of the 100 participants, 90% were aged 65 years and older. Common comorbid medical conditions included hypertension (21%), arthritis (13%), diabetes (11%), sleep apnea (9%), depression (7%), stroke (5%), glaucoma (5%), and Parkinson's disease (4%). At the time of assessment, 67% of the sample had a valid driver's license, while 18% had a 90-day suspended license, 14% had a temporary license, and 1% had a beginner's license. Participants had been driving for a mean of 57.2 years (*SD*  =  9.6, range 29–73).

### Clinical Scores

Overall, 70% of the sample took 3 or more minutes, with 45.5% taking 5 or more minutes to complete the Trails B. Participants’ performance on the UFOV varied, with 27.3% having a Risk Index rating between 1 (very low risk) and 2 (low risk), and 72.7% having a Risk Index rating between 3 (low to moderate) and 5 (very high risk).

Table 1 shows the clinical test scores between those who passed (44%) and failed (56%) the on-road assessment. Participants who failed the on-road assessment were significantly older, drove for more years, were more likely to have a grade 12 or lower education, and scored poorer on the MoCA, Trails B, and the UFOV subtests. Those who failed the on-road assessment were also significantly more likely to have a UFOV Risk Index of 5, indicating a very high risk for driving impairment.

**Table 1:** Comparisons of Older Drivers with CI who Passed Versus Failed the On-Road Assessment.

Variables	Total (*N* = 100)	Pass (*n* = 44)	Fail (*n* = 56)	Significance (*p* < .05)
Demographics				
Age	76.2 (8.8) 45–94	71.6 (9.8) 45–88	79.8 (6.0) 60–94	*t* = −4.9, *p* < .001
Gender				
Male	79 (79.0%)	37 (84.1%)	42 (75.0%)	*χ^2^* = 1.2, *p* = .27
Female	21 (21.0%)	7 (15.9%)	14 (25.0%)	
Education				
More than grade 12	52 (53.1%)	28 (65.1%)	24 (43.6%)	*χ^2^* = 4.5, *p* = .03
Grade 12 or lower	46 (46.9%)	15 (34.9%)	31 (56.4%)	
Location				
Ontario	79 (79%)	37 (84.1%)	42 (75.0%)	*χ^2^* = 1.2, *p* = .27
Saskatchewan	21 (21%)	7 (15.9%)	14 (25.0%)	
Driving history				
License status				
Valid	66 (67%)	29 (67.4%)	37 (66.1%)	*χ^2^* = 1.5, *p* = .69
90-day suspension	18 (18%)	7 (16.3%)	11 (19.6%)	
Temporary	14 (14%)	6 (14.0%)	8 (14.3%)	
Beginner's	1 (1%)	1 (2.3%)	0 (0%)	
Number of years driving	57.2 (9.6) 29–73	53.5 (10.1) 29–71	60.2 (8.2) 30–73	*t* = 3.6, *p* < .001
Clinical tests				
MoCA (0–30)	18.9 (4.4) 6–25	20.1 (4.4) 6–25	18.0 (4.1) 8–25	*t* = 2.5, *p* = .01
Trails B time for completion (min)	4.58 (2.41) 0.57–13.06	4.16 (2.46) 0.57–11.25	5.33 (2.29) 1.37–13.06	*t* = −2.3, *p* = .02
<3.00 min	26 (30%)	18 (45.0%)	8 (16.7%)	χ^2^ = 8.4, *p* = .004
≥3.00 min	62 (70%)	22 (55.0%)	40 (83.3%)	
<5.00 min	48 (54.5%)	27 (67.5%)	21 (43.7%)	χ^2^ = 5.0, *p* = .03
≥5.00 min	40 (45.5%)	13 (32.5%)	27 (56.3%)	
UFOV subtest 1 (ms)	92.8 (144.6) 9–500	58.5 (114.4) 9–500	120.9 (161.0) 9–500	*t* = −2.2, *p* = .03
UFOV subtest 2 (ms)	307.2 (181.7) 9–500	228.5 (177.7) 9–500	372.5 (159.0) 9–500	*t* = −3.9, *p* < .001
UFOV subtest 3 (ms)	408.6 (131.4) 16–500	353.6 (137.3) 83–500	456.0 (106.4) 16–500	*t* = −3.7, *p* < .001
UFOV Risk Index				*χ^2^* = 17.2, *p* = .002
1	13 (14.8%)	10 (25.6%)	3 (6.1%)	
2	11 (12.5%)	8 (20.5%)	3 (6.1%)	
3	12 (13.6%)	5 (12.8%)	7 (14.3%)	
4	21 (23.9%)	10 (25.6%)	11 (22.4%)	
5	31 (35.2%)	6 (15.4%)	25 (51.0%)	

*Note.* MoCA = Montreal Cognitive Assessment; Trails B = Trail Making Test Part B; UFOV = Useful Field of View®; CI =cognitive impairment. Variables are presented as mean (*SD*) and range for continuous data and frequencies (percentages) for categorical data. Number of participants (*n* = 100) except for education (*n*=98), license status (*n* = 99); number of years driving (*n* = 95); Trails B (*n* = 88); UFOV subtest 1 (*n* = 91); UFOV subtest 2 (*n* = 86); UFOV subtest 3 (*n* = 82); UFOV Risk Index (*n* = 88).

Table 2 shows the correlations between age and clinical measures. Almost all clinical measures were significantly correlated with each other; except there was no significant association between the Trails B and UFOV subtest 1. The UFOV Risk Index was significantly correlated with age and all clinical measures. Age was significantly correlated with the UFOV subtests 2 and 3 and the Risk Index.

**Table 2:** Correlations Between Age and Clinical Test Scores.

	Age	MoCA	Trails B	UFOV Subtest 1	UFOV Subtest 2	UFOV Subtest 3
Age	—	—	—	—	—	—
MoCA	−.11	—	—	—	—	—
Trails B	.20	−.50**	—	—	—	—
UFOV Subtest 1	.16	−.43**	.20	—	—	—
UFOV Subtest 2	.26**	−.55**	.57**	.54**	—	—
UFOV Subtest 3	.55**	−.36**	.55**	.37**	.58**	—
UFOV Risk Index^1^	.42**	−.56**	.60**	.53**	.83**	.77**

*Note*. MoCA = Montreal Cognitive Assessment; Trails B = Trail Making Test Part B; UFOV = Useful Field of View®. Pearson's bivariate correlations. ^1^Rank biserial correlations. Number of participants (*n* = 100) except for Trails B (*n* = 88); UFOV subtest 1 (*n* = 91); UFOV subtest 2 (*n* = 86); UFOV subtest 3 (*n* = 82); UFOV Risk Index (*n* = 88). *p < .05, **p < .01.

### Receiver Operating Contrast Characteristic Curves

**Figure 1.**
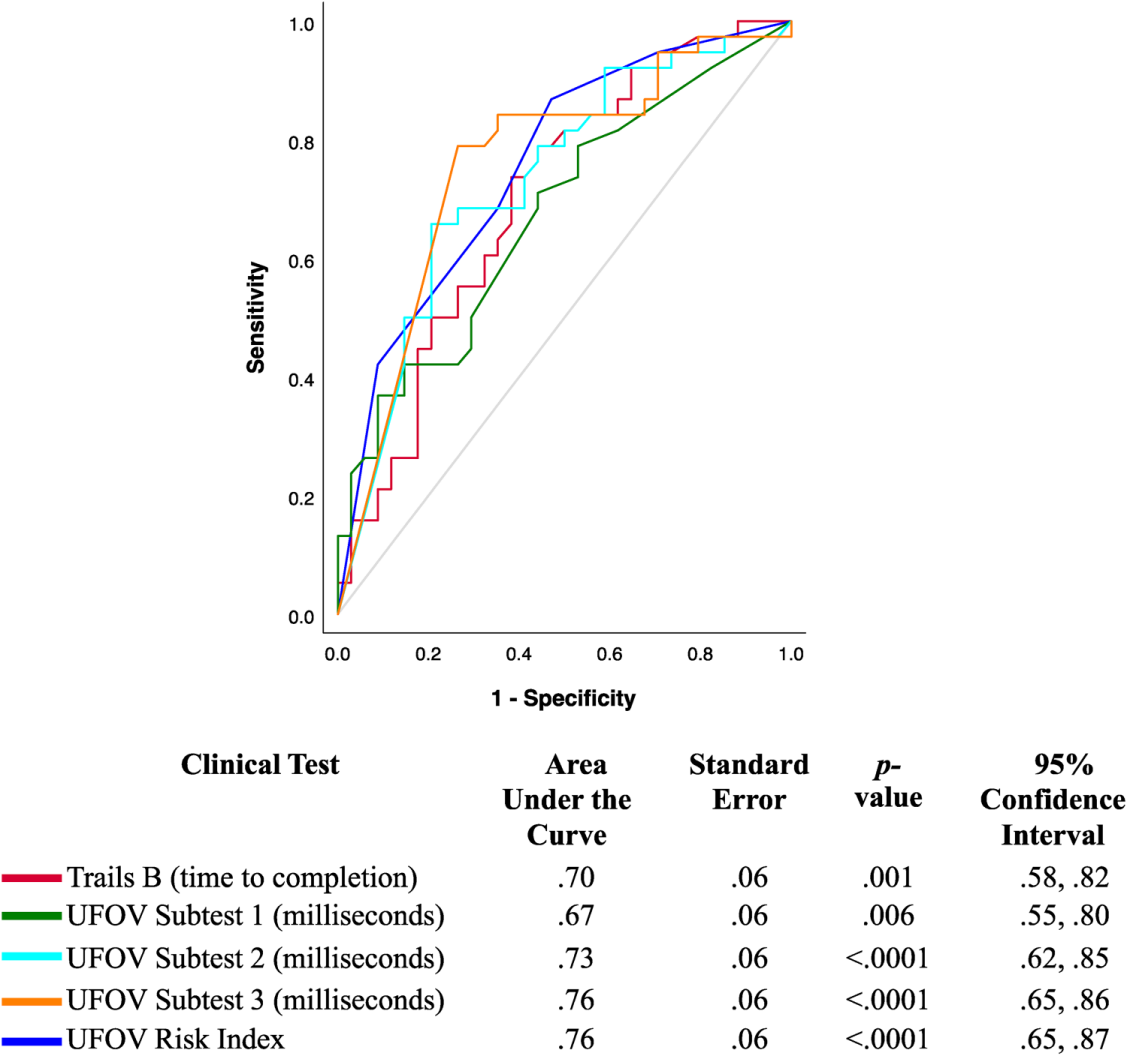
The UFOV and Trails B Predicting Pass Versus Fail Outcomes of an On-road Assessment in Older Drivers with CI (*N*  =  72, 34 Passed, 38 Failed).

*Note*. Trails B = Trail Making Test Part B; UFOV = Useful Field of View^®^; CI = cognitive impairment.

Figure 1 presents the ROC curves plotting the predictive validity for the Trails B, UFOV subtests, and the UFOV Risk Index in predicting pass/fail outcomes on the on-road assessment. Except for the UFOV subtest 1 (AUC = .67, *p* = .006), the Trails B (AUC = .70, *p* = .001), UFOV subtest 2 (AUC = .73, *p* < .0001), UFOV subtest 3 (AUC = .76, *p* < .0001), and UFOV Risk Index (AUC = .76, *p* < .0001) had moderate accuracy in detecting on-road outcomes. When compared against one another, there were no significant differences between the Trails B and the UFOV subtest 1 (*z* = .36, *p* = .72, AUC difference = .03, SE difference = .35, 95% CI = [−.12, .18]), UFOV subtest 2 (*z* = −.53, *p* = .60, AUC difference = −.03, SE difference = .34, 95% CI = [−.15, .09]), UFOV subtest 3 (*z* = −.99, *p* = .32, AUC difference = −.06, SE difference = .34, 95% CI = [−.17, .06]), or the UFOV Risk Index (*z* = −1.03, *p* = .31, AUC difference = −.06, SE difference = .34, 95% CI = [−.16, .05]) in predicting pass/fail on-road outcomes.

Table 3 presents the optimal cut-points of the Trails B, UFOV subtests, and UFOV Risk Index that predicted pass/fail outcomes on the on-road assessment. On the Trails B, a cut-point ≥3.58 min optimally predicted pass/fail outcomes with a sensitivity of 73.7% and a specificity of 61.8%, but misclassified 31.7% of those who passed (i.e., false positives) and 32.3% of those who failed (i.e., false negatives) the on-road assessment. These findings resulted in balanced positive (68.3%) and negative predictive values (67.7%) but a high error rate (64.5%). On the UFOV, a cut-point ≥467 ms on subtest 3 optimally predicted pass/fail outcomes with a sensitivity of 78.9% and a specificity of 73.5%. The false positive rate was 23.1% and the false negative rate was 24%. These findings also resulted in a balanced positive of 76% with negative predictive values of 75.8% and an error rate of 47.6%.

**Table 3:** Cut-Points of Clinical Tests for Predicting On-Road Outcomes in Older Drivers with CI (*N* = 72).

Clinical Test Cut-Point	Sensitivity	Specificity	Predictive Value		Misclassified	No. False		Error Rate	Youden's Index
			+	−		+	−		
Trails B									
3.58 min	73.7% (28/38)	61.8% (21/34)	68.3% (28/41)	67.7% (21/31)	31.9% (23/72)	13	10	64.5%	35.5%
3.00 min	81.6% (31/38)	50.0% (17/34)	64.6% (31/48)	70.8% (17/24)	33.3% (24/72)	17	7	68.4%	31.6%
UFOV subtest 1									
64.5 ms	36.8% (14/38)	91.2% (31/34)	82.4% (14/17)	56.4% (31/55)	37.5% (27/72)	3	24	72.0%	28.0%
UFOV subtest 2									
276.5 ms	65.8% (25/38)	79.4% (27/34)	78.1% (25/32)	67.5% (27/40)	27.8% (20/72)	7	13	54.8%	45.2%
UFOV subtest 3									
467 ms	78.9% (30/38)	73.5% (25/34)	76.9% (30/39)	75.8% (25/33)	23.6% (17/72)	9	8	47.6%	52.4%
UFOV Risk Index									
≥2 (vs 1)	94.7% (36/38)	29.4% (10/34)	60.0% (36/60)	83.3% (10/12)	36.1% (26/72)	24	2	75.9%	24.1%
≥3 (vs <3)	86.8% (33/38)	52.9% (18/34)	67.3% (33/49)	78.3%(18/23)	29.2% (21/72)	16	5	60.3%	39.7%
≥4 (vs <4)	68.4% (26/38)	64.7% (22/34)	68.4% (26/38)	64.7% (22/34)	33.3% (24/72)	12	12	66.9%	33.1%
5 (vs <5)	42.1% (16/38)	91.2% (31/34)	84.2% (16/19)	58.5% (31/53)	34.7% (25/72)	3	22	66.7%	33.3%

*Note*. Trails B = Trail Making Test Part B; UFOV = Useful Field of View®; min  =  minutes to test completion; ms = milliseconds. Sensitivity is the proportion of those with a clinical test score that is equal to or greater than the cut-point out of all who failed. Specificity is the proportion of those with a clinical test score that is less than the cut-point out of all who passed. The analysis includes the 72 participants who completed all clinical tests, that is, Trails B, UFOV subtests 1–3, and Risk Index, and has a pass/fail outcome.

## Discussion

The findings show that the Trails B (time for completion) and UFOV subtest 2, subtest 3, and Risk Index displayed moderate discriminability for predicting pass/fail outcomes (e.g., AUC ≥ .70, *p* ≤ .05). Overall, the moderate predictive ability, sensitivity, and specificity of <80% and the high error rates show that the Trails B and UFOV performed sub-optimally for predicting pass/fail on-road outcomes in older drivers with CI. Compared to other studies, the Trails B and UFOV did not perform as well in our sample of older drivers with CI. While all participants in our sample had CI, between 23% and 48% of participants in other studies had CI (Bowers et al. 2013; Dobbs & Shergill, 2013; Duncanson et al., 2018; Stern et al., 2016), or the study included only healthy older drivers with no CI (Classen et al., 2013). Furthermore, our sample performed poorer on the MoCA compared to prior studies (e.g., Bowers et al., 2013). Additionally, although we cannot make direct comparisons, prior studies used the Mini-Mental State Exam (MMSE) to identify CI with scores ranging from 25.3 to 28 (e.g., Classen et al., 2013; Dobbs & Shergill, 2013; Stern et al., 2016), indicating the samples in these studies did not have severe CI. Lastly, the MoCA is more sensitive in detecting MCI/CI compared to the MMSE (De Roeck et al., 2019). The high degree of CI in the present study likely accounts for the poor sensitivity and specificity of the Trails B and UFOV subtests and Risk Index.

On average, our sample of participants completed the Trails B in 4.58 min, with scores varying between 57 s and 13.06 min. Our scores were poorer than reported in prior studies of healthy older drivers and those with CI, who took between 102.1 and 148.8 s (Classen et al., 2013; Dobbs & Shergill, 2013; Duncanson et al., 2018) and 166.4 and 179.1 s (Dobbs et al., 2013; Duncanson et al., 2018), respectively. Compared to the ≥3.00 min cut-point (Roy & Molnar, 2013), our cut-point of ≥3.58 had poorer sensitivity (73.7% vs 81.6%) but higher specificity (61.8% vs 50.0%), slightly higher positive predictive values (68.3% vs 64.6%), and lower negative predictive values (67.7% vs 70.8%). The lower sensitivity and higher positive predictive values of the Trails B cut-point ≥3.58 min (vs ≥3.00 min) likely occurred due to more participants in our study taking longer than 3 min to complete the Trails B (70% vs 30%) despite only 56% failing the on-road assessment. Notably, 70% took 3 min or longer with 45.5% taking 5 min or longer to complete the Trails B. These findings are consistent with findings by Papandonatos et al. (2015) who found that more than 25% of older drivers with CI did not complete the Trails B within the allotted time (i.e., 5 min). Furthermore, those who took <3.58 min (vs 3.00 min) were more accurately identified as passing the on-road assessment (specificity, 61.8% vs 50.0%). Based on our findings, the Trails B would fail most older drivers with CI whether they are fit or unfit to drive. Accordingly, the Trails B may be too challenging of a test for some older drivers with CI and may not be useful for identifying those who are unfit to drive.

On the UFOV, our sample of participants had scores that ranged from 9 to 500 ms on the subtests, with mean scores of 92.8, 307.2, and 408.6 for subtests 1, 2, and 3, respectively; 72.7% had a Risk Index between 3 and 5 indicating “moderate” to “very high” risk for driving impairment. Similarly, when compared to other studies with healthy and CI drivers, our sample performed poorer on the UFOV subtests and Risk Index. For example, UFOV subtest 1 scores have ranged from 23.8 and 58.5 ms (Bowers et al., 2013) and 123.9 and 360 ms on subtest 2 (Bowers et al., 2013; Stern et al., 2016). Classen et al. (2013) found that healthy older drivers completed the UFOV with a mean score of 32.6 ms on subtest 1, 123.9 ms on subtest 2, and 269.9 ms on subtest 3; only 28.5% had a Risk Index between 3 and 5 (Classen et al., 2013), indicating a sample with a significantly lower risk for driving impairment. Furthermore, our cut-point of ≥467 ms on subtest 3 optimally predicted pass/fail outcomes in older drivers with CI, showing that our sample performed worse on the UFOV subtests than those in other studies (Bowers et al., 2013; Classen et al., 2013). For example, Bowers et al. (2013) found that ≥191.5 ms on UFOV subtest 2 best predicted safe versus at-risk older drivers (11 of 47 drivers had MCI). Similarly, Classen et al. (2013) found that a cut-point ≥106.6 ms on the UFOV subtest 2 predicted pass/fail outcomes in healthy older drivers with 88% sensitivity, 63% specificity, 32% positive predictive value, 96% negative predictive value, and 49% error rate); however, the UFOV Risk Index of ≥3 predicted pass/fail outcomes with the lowest error (81% sensitivity, 81% specificity, 46% positive predictive value, 96% negative predictive value, and 38% error rate) (Classen et al., 2013). These studies included healthier samples of older drivers (Bowers et al., 2013; Classen et al., 2013; Stern et al., 2016), which resulted in better performance on the UFOV and cut-points that reflected better scores in visual processing speed and divided attention than in our sample of older drivers with CI who experienced more difficulty on the subtests, scored poorer, and thus, had cut-points that reflected impaired visual processing speed, divided attention, and selective attention.

The associations between age and clinical test scores further highlight the level of CI in the sample, which comprised mostly of older drivers (90%). Furthermore, those who failed were significantly older than those who passed. Given that age was not associated with MoCA scores (indicating that our sample had significant cognitive deficits regardless of age), it is likely that more severe aging-related deficits (e.g., slower reaction time) contributed to those who failed the CDE. However, since clients referred for CDEs are often individuals who may be experiencing a change in CI that may be typical given their age, it is not always possible to isolate the proportion of age-related deficits from CI that contribute to a person's fitness to drive. Accordingly, we did not control for age in any analyses in this study.

Poorer MoCA scores were significantly correlated with poorer scores on the Trails B and all UFOV tests (including the Risk Index) suggesting that impaired cognition (as per the MoCA) was associated with poorer processing speed (UFOV subtest 1), divided (Trails B, UFOV subtest 2), and selective attention (UFOV subtest 3). The association between scores on the Trails B and UFOV indicates the commonalities in the visual-cognitive functions tested (Woutersen et al., 2017). For example, the UFOV subtests 2 and 3, which measure divided and selective attention, respectively, both correlated with the Trails B. Since the UFOV subtest 1 did not correlate with age or Trails B, nor did it show acceptable predictive validity for pass/fail outcomes (i.e., AUC <.70), subtest 1 may not be challenging enough to identify at-risk older drivers with CI. As Trails B requires executive function, as well as visual processing speed and divided/selection attention, the test may be more challenging to complete than the UFOV subtests 2 and 3, which may be why most participants in this study took more than 3 min to complete the Trails B. The UFOV subtests 2 and 3, which test a person's divided attention and selective attention individually, may be not as challenging as the Trails B to complete and may be more suitable to identify deficits in skills required to drive safely in older drivers with CI.

### Study Limitations

Data was collected retrospectively and although we could identify participants with CI based on MoCA scores, we did not have a confirmed diagnosis of CI (e.g., MCI, Alzheimer's disease, and dementia) or information about the symptomatology that can impair driving performance. Consequently, our findings may not be generalized to those with specific diagnoses and severities of CI, although the participants in our study are typical of those seen within driving assessment clinics (i.e., with the heterogeneity of CI symptoms and severity) (Vrkljan et al., 2013). Additionally, as participants were referred for CDEs, performed by occupational therapists trained in assessing fitness to drive (i.e., certified driver rehabilitation specialist, graduate certificate in assessing driving capabilities), our findings are not generalizable to primary care settings where CI is not as prevalent or as severe. The CDE data did not include information on medications prescribed to participants. Accordingly, we could not examine whether medications (or side effects) could have contributed to participants’ performance on the CDE. Moreover, the Trails B and UFOV may not be complex enough to identify deficits outside of divided attention and information processing speed given that driving is a multifaceted complex task that requires the integration of multiple cognitive domains. Additionally, the process of how pass/fail decisions were determined may have differed between driving assessment centers. Nevertheless, the occupational therapists made these determinations (which were not significantly different between locations) based on participants’ on-road driving performance and whether they completed 50% of total maneuvers correctly. Moreover, 28 participants did not complete all clinical tests, which reduced our sample size for the ROC curve analyses from 100 to 72, which may have reduced the accuracy of the tests’ ability to predict pass/fail outcomes. Examining the predictive ability of the Trails B and UFOV with a larger sample may confirm the accuracy of the tests’ ability to predict on-road outcomes for older drivers with CI.

### Implications for Practice

Our study findings show that Trails B and UFOV do not perform as well in drivers with more severe CI. As such, we recommend occupational therapists consider the following implication to guide their practice:

- Screening for CI, particularly divided and selective attention, is important for identifying medically at-risk older drivers with CI. Occupational therapists should consider using tests that measure various visual-cognitive functions (e.g., visual processing speed, visual scanning, divided/selective attention, and executive functioning) such as the UFOV subtests 2 and 3. Although the tests can be used to identify deficits in visual attention, neither test should be solely used to make decisions concerning medical fitness to drive.

## Conclusion

Overall, we conclude that the Trails B and UFOV performed only moderately well when used to identify drivers with CI who were referred by physicians for a CDE to determine their medical fitness to drive. As 70% of drivers included in this study took 3 or more minutes to complete the Trails B, the test may be too challenging for older drivers with CI who have been referred for a CDE thereby wrongly identifying those who are fit to drive as unfit or vice versa. A cut-point of 467 ms on the UFOV subtest 3 provided the lowest error rate. The UFOV subtest 3 may be more useful than the Trails B for predicting pass/fail outcomes in drivers with more severe CI who are referred for CDEs.

## Key Messages

- The UFOV subtests 2 and 3 can identify older drivers with CI who have impaired behind-the-wheel performance, but should not solely be used to determine fitness to drive.
- The Trails B may be too challenging for some older drivers with CI, and, as such, may wrongly identify those who are medically fit to drive as unfit, or vice versa.
